# Margarine versus Butter

**Published:** 1916-02-19

**Authors:** 


### Margarine versus Butter.

The Wandsworth Board of Guardians, following the lead of many other public bodies, has decided to supply the patients in their infirmaries and the officials with margarine in place of butter. The board was influenced in its decision by a report from Mr. A. H. Muter, Public Analyst for Lambeth and Wandsworth, who reported that there was very little difference in the amount of fatty matter in and the assimilability of the two substances. A comparison of the two is complicated by the fact that the word "margarine" is applied to many substances of different composition. As defined by the Margarine Act of 1887, margarine is the name given to "all substances, whether compounds or otherwise, prepared in imitation of butter, and whether mixed with butter or not." Margarine to-day is made chiefly of vegetable fats, such as cocoanut oil and palm-kernel oil, mixed with separated milk, and artificially coloured. The result is a product which in the case of the best margarines cannot easily be distinguished, either by the eye or by the palate, from butter. As to its value as a food, there is a sharp division amongst medical authorities. Dr. James Oliver says that vegetable fats are less digestible than milk fat, and that they deprive the body of calcium salts by forming in the intestines calcium soaps, which cannot readily be utilised. Butter is also said to contain phosphorised fats which margarine does not. On the other hand, Dr. Robert Hutchison asserts that 102 pounds of margarine are equal in nutritive value to 100 pounds of butter, and that it is practically as well absorbed as butter, but there is no deprivation of calcium, because calcium is always in excess in any dietary, and that the amount of phosphorus in butter is negligible. Another objection that has been raised is that, owing to its method of manufacture, there are no vitamines present in margarine, whilst butter contains them. The advocates of margarine point out, however, that vitamines can easily be supplied in other articles of diet. Most medical men will agree that the opponents of margarine fail to prove the essential superiority of butter, and that, having regard to the greater cheapness of margarine, its substitution for butter in institutions is perfectly justified. The latter statement, however, requires three qualifications—the margarine supplied must be of the best quality, the medical officer must have power to order butter in special cases, and the dietary must be of such a character as to ensure the supply of an excess of calcium salts and the presence of vitamines.

